# Cutaneous squamous cell carcinoma (SCC) arising in stump of amputated finger in a patient with resected glossal SCC

**DOI:** 10.1186/1756-0500-5-595

**Published:** 2012-10-30

**Authors:** Kenzo Uchida, Tsuyoshi Miyazaki, Hideaki Nakajima, Kohei Negoro, Daisuke Sugita, Shuji Watanabe, Mitsuo Yoshimura, Hiroshi Itoh, Hisatoshi Baba

**Affiliations:** 1 Department of Orthopaedics and Rehabilitation Medicine, Faculty of Medical Sciences, University of Fukui, Matsuoka Shimoaizuki 23-3, Eiheiji, Fukui, 910-1193, Japan; 2 Tumor Pathology, Faculty of Medical Sciences, University of Fukui, Matsuoka Shimoaizuki 23-3, Eiheiji, Fukui, 910-1193, Japan

**Keywords:** Squamous cell carcinoma, Finger amputation stump, Glossal tumor, Double cancer

## Abstract

**Background:**

Cutaneous squamous cell carcinoma (SCC) of the hands and fingers are sometimes locally aggressive; with higher rates of regional metastasis than other cutaneous SCC, although distant metastasis is rare.

**Case presentation:**

We present the case of a 62–year-old Japanese man with double cancers: a tongue SCC and a cutaneous SCC. Swelling of the finger lesion developed gradually around the entire remaining middle finger after accidental amputation at the proximal interphalangeal joint. Histopathological examination of the tumor on the stump of the amputated finger indicated a well-differentiated SCC. The past history indicated surgery for SCC of the tongue 3 years earlier; with histopathology of moderately-differentiated SCC.

**Conclusion:**

Since dedifferentiation is unlikely in metastatic tumors, the cutaneous SCC of the finger is unlikely to have originated from the tongue SCC. Alternatively, the double cancer may be two unrelated lesions or the tongue tumor could have originated from the cutaneous SCC.

## Background

Post-traumatic cutaneous squamous cell carcinoma (SCC, also known as Marjolin’s ulcer) arising in chronic scars, ulcers and sinuses is not common [1-3]. The development of a scar or ulcer neoplasm is considered rare, with incidence among all SCC of 2% [4]. Distant metastasis is also extremely rare [5,6]. We here report a case of cutaneous SCC arising in an amputated finger stump in a patient with tongue SCC resected 3 years earlier. We also discuss the relation between the origins of the cutaneous SCC and glossal SCC.

## Case report

**Figure 1:**
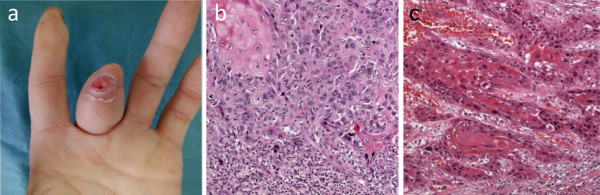
**(a) Macroscopic appearance.** The tumor is on the tip of the left middle finger with deep ulceration at the center of the operation scar. (**b**) Histopathology of the tumor of the middle finger resected in 2010 showing well-differentiated SCC. Cancer cells have enlarged hyperchromatic nuclei and proliferate in verrucous structures or solid nests with apparent cancer pearls (hematoxylin and eosin staining, original magnification ×40). (**c**) Histopathology of the tongue tumor resected in 2007. Cancer cells show individual keratinization without definitive cancer pearls, indicating moderately-differentiated SCC (hematoxylin and eosin staining, original magnification ×40).

In 1985, a 37-year-old Japanese man suffered accidental amputation of the phalanx of the left middle finger by an electric saw. The stump of the amputated finger at the proximal interphalangeal joint subsequently healed without any infection. Ten years later, the stump tip started to swell gradually without apparent cause; and progressively increased in size over the following 10 years, around the entire remaining middle finger. However, the patient did not seek medical advice since the growth was never painful. Twenty-two years after the accident, the patient consulted an otorhinolaryngologist for an ulcerative lesion on the left lower aspect of the tongue. The entire glossal lesion was resected one month later. The ulcer size was approximately 1.5 × 1.5 cm and histopathological examination established the diagnosis of moderately-differentiated SCC with individual cell keratinization (Figure 1c), pT1N0M0, Stage I.

By 2010, the cutaneous lesion in the middle finger had increased in size gradually over the preceding 3-month period, including ulceration with discharge (Figure 1a). Histopathological examination of a biopsy taken by the local physician showed a well-differentiated SCC. The patient was immediately referred to our University Hospital for further management and was admitted. No signs of destruction in the bony proximal phalanx of the middle finger were evident on plain X-ray and MRI. The lesion was resected widely *en bloc* through amputation of the corresponding MP joint; according to the Clinical Practice Guideline for Squamous Cell Carcinoma by the Japanese Dermatological Association. The final pathological diagnosis was well-differentiated SCC (Figure 1b). The resected tissue margins were free from cancer cells. The patient has been doing well for more than 1 year after surgery. A new whole-body FDG-PET examination showed no recurrence or metastasis during the 1-year postoperative follow-up.

## Discussion

Cutaneous SCC, a malignant lesion that originates from epidermal keratinocytes, is composed of malignant keratinocytes with the capacity for metastatic spread, and is a well-recognized complication of chronic scars, ulcers and sinuses [2]. In addition, Marjolin’s ulcer is also known as post-traumatic skin SCC, which presents as an aggressive malignant lesion in skin zones that have withstood chronic irritation [1,3]. The latency period between the initial injury and malignant transformation varies from 20 to 30 years in most cases of Marjolin’s ulcers [7,8]. Cutaneous SCC and other tumors of the skin, either primary or secondary, initially present as inflammatory conditions of the fingers with areas of ulceration. Our case is unusual in that it is an example of SCC arising in a scar over a 10-year period after amputation stump surgery of the finger.

Metastatic lesions in the hands and fingers are rare. About two thirds of the reported metastatic tumors of the hand and finger originated from lung and breast tumors; while the remaining 35% originated from the gastrointestinal tract, prostate, kidney, parotid gland, skin, and nasopharynx [9]. Histopathologically, the metastasis closely resembles the primary lesion, although it can be less differentiated [10]. On the other hand, histopathology of the skin biopsy of this case indicated a well-differentiated SCC. Development of metastatic lesions in the hands is associated with diffuse tumor spread and poor prognosis; and the mean survival is a few months only [11]. Only a few cases have been reported, where metastasis appeared in the soft tissues of the distal digits, with underlying bone being only secondarily involved [12]. Furthermore, the reported incidence of skin metastasis from SCC of the head and neck ranges from 0.8% to 1.3% [13], with most cases being sporadic. Interestingly, two cases with neck skin secondaries from supraglottis carcinoma T1N0, Stage I, similar to our case, had been reported previously [14]. Was the skin tumor in our case a metastatic lesion from the tongue tumor that was excised 3 years earlier? or was it the primary lesion whereas the tongue tumor was secondary? or were the tumors two separate entities? Histopathological examination of the middle finger lesion showed a well-differentiated SCC while that of the tongue was a moderately-differentiated SCC. Since dedifferentiation is unlikely in metastatic process, the cutaneous SCC of the finger in this case is unlikely to have originated from the glossal SCC. In our patient, the finger lesion developed gradually, and progressively increased in size over a 10-year period around the whole remaining middle finger. This is a possible case of double cancer: a cutaneous SCC and glossal SCC. While a double cancer may have developed simultaneously in our patient, the probability of metastasis to the tongue originating from the cutaneous well-differentiated SCC of the amputation stump of the finger, cannot be ruled out.

In some series, SCC of the hands and fingers were reported to be more locally aggressive; with higher rates of regional and distant metastases, than other cutaneous SCC [5]. Most cases are invasive, involving the bone or draining lymph nodes. Distant metastasis is rare [6]. Previous reports described a metastasis rate of 10% for cutaneous SCC [15] with a 3-year survival rate of 94% for well-differentiated posttraumatic SCC [16].

## Conclusion

We presented the case of a patient with two anatomically separate tumors with a question mark regarding their origins. Perhaps recent molecular pathological techniques, such as comparative genomic hybridization, could be employed in the future to determine the cellular origin of double cancers of similar histopathology.

## Consent section

Written informed consent was obtained from the patient for publication of this manuscript and accompanying images. A copy of the written consent is available for review by the Editor-in-Chief of this journal.

## Competing interests

The authors declare no conflict of interest.

## Authors' contributions

KU drafted the manuscript and was the main surgeon in charge. TM, KN, DS and SW were part of the surgical team and responsible for the patient postoperative care. HN, MY and HI revised the manuscript and provided medical support. HB participated in the conceptualization and final version of the manuscript. All authors read and approved the final manuscript.
